# Learning experience facilitates sparse coding of new odors in a large-scale olfactory bulb model

**DOI:** 10.1186/1471-2202-16-S1-P297

**Published:** 2015-12-18

**Authors:** Shanglin Zhou, Boqiang Fan, Michele Migliore, Yuguo Yu

**Affiliations:** 1 School of Life Sciences, the State Key Laboratory of Medical Neurobiology and Institutes of Brain Science, Fudan University, Shanghai, 200433, China; 2 Institute of Biophysics, National Research Council, 90146 Palermo, Italy

## 

**Figure 1:**
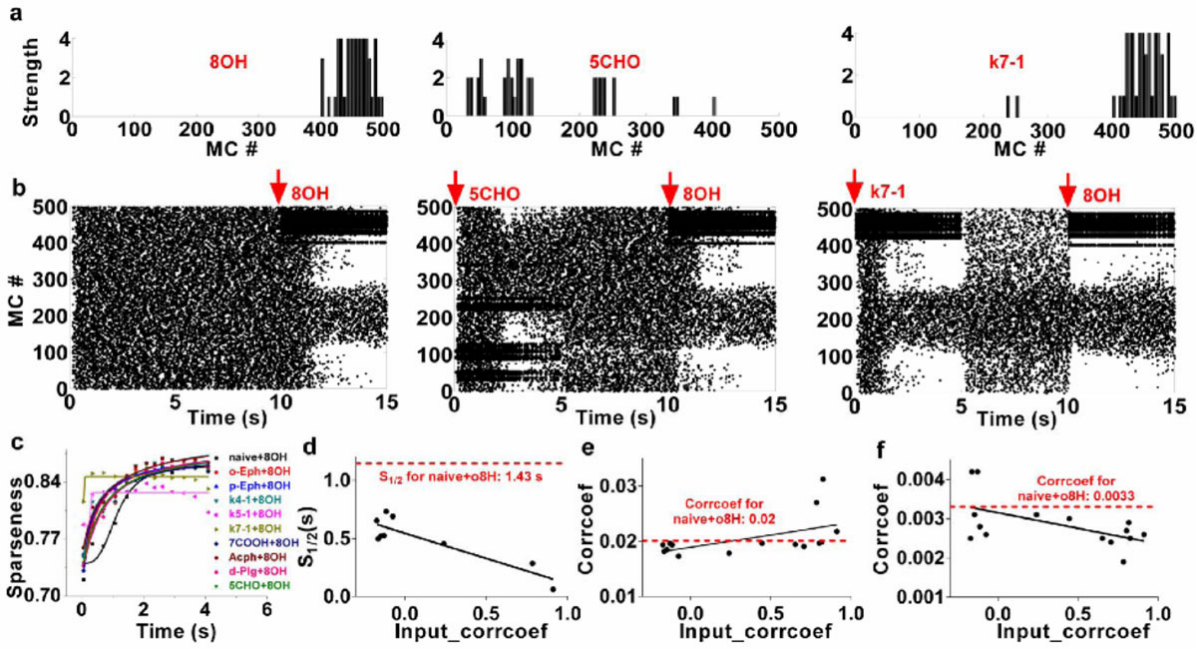
**(a) odor inputs 8OH, 5CHO and k7-1**. (b) MC network response to odor 8OH for naïve training (left), 5CHO prior training (middle) and k7-1 training (right). (c) Network sparseness develops for different sets of prior odor training. (d) Sparseness onset time S_1/2 _vs. input signal correlation coefficient (corrcoef.). (e) Mean corrcoef between MCs within the network vs. input signal corrcoef. (f) Response similarity (quantified by corrcoef between MC network responses to different odor inputs) vs input signal corrcoef.

Odor responses of mitral cells in the olfactory bulb are observed to be sparse spatially and decorrelated in response to odor signals [1]. We have built a large scale biophysical network model of olfactory bulb composed of mitral and granule cells, corresponding to 1/100th of the real system in the rat, and used direct experimental imaging data of glomeruli activated by various odors. Our previous reports have showed that a sparse spatial spiking representation of specific odor signals can emerge naturally within several seconds learning period from the mitral-granule cell interactions, realistically implemented in our model with self-organizing dendrodendritic synapses driven by mitral cell activity [2]. To address how the prior odor experience interfere with subsequent sparse coding of new coming odors, we trained the network with a set of odors (Figure 1a and 1b, note that no signal in left panel of Figure 1b which is called native state) to reach a stable response state (sniff frequency around 4-10 Hz) within 5 seconds (Figure 1a), and then a second odor (e.g., octanol 8OH) was input to the network (5s, 4-10 Hz) (Figure 1b, middle and right). Compared to the network trained from the native state (Figure 1b, left), prior active odor training could significantly facilitate the whole mitral cell network into different degrees of response sparseness for odor 8OH (see right two panels in Figure 1b). The sparseness onset time S1/2 (defined as the time reached the half of maximum sparseness) is relatively large for input signals with less similarity (Figure 1c) while become shorter as more input similarity is induced (Figure 1d, correlation coefficient r = 0.89), however, all shorter than that in native situation (Figure 1c, d), suggesting prior training experience could significantly promote odor perception of even novel odors.

Furthermore, for the most of total 16 prior odor training tests, the mean correlation coefficients between MT cells within the network measured at the end of after-learning process (i.e. No.14-15s) are less than the native situation (Figure 1e), suggesting a more decorrelated state. Correspondingly, the similarity of the MT network responses for most of the tests is lower significantly than the naïve case (Figure 1f). This strongly suggests prior odor experience could significantly promote odor discrimination ability of the olfactory bulb network. In sum, odor experience could accelerate the formation of network response sparseness, decorrelation, and promote signal discrimination.
